# Anti-Inflammatory Effect of *Momordica Charantia* in Sepsis Mice

**DOI:** 10.3390/molecules190812777

**Published:** 2014-08-21

**Authors:** Che-Yi Chao, Ping-Jyun Sung, Wei-Hsien Wang, Yueh-Hsiung Kuo

**Affiliations:** 1 Department of Health and Nutrition Biotechnology, Asia University, Taichung, 41354, Taiwan; 2 Graduate Institute of Marine Biotechnology and Department of Life Science and Institute of Biotechnology, National Dong Hwa University, Pingtung 94450, Taiwan; E-Mail: pjsung@nmmba.gov.tw; 3 National Museum of Marine Biology and Aquarium, Pingtung 94450, Taiwan; E-Mail: whw@mail.nsysu.edu.tw; 4 Department of Marine Biotechnology and Resources, National Sun Yat-sen University, Kaohsiung 80424, Taiwan; 5 Department of Chinese Pharmaceutical Sciences and Chinese Medicine Resources, China Medical University, Taichung 40402, Taiwan; 6 Department of Biotechnology, Asia University, Taichung 41354, Taiwan

**Keywords:** *Momordica charantia*, LPS, sepsis, anti-inflammation, PPARs

## Abstract

Wild bitter gourd (*Momordica charantia* L. var. *abbreviate* Seringe), a common vegetable in Asia, is used in traditional medicine to treat various diseases, including inflammation. Extant literature indicates that wild bitter gourds have components that activate PPARα and PPARγ. This research probed the influence of adding wild bitter gourd to diets on inflammation responses in mice with sepsis induced by intraperitoneal injection of LPS. Male BALB/c mice were divided normal, sepsis, positive control, and three experimental groups. The latter ate diets with low (1%), moderate (2%), and high (10%) ratios of wild bitter gourd lyophilized powder. Before mice were sacrificed, with the exception of the normal group, intraperitoneal injection of LPS induced sepsis in each group; positive control group was injected with LPS after PDTC. This experiment revealed starkly lower weights in groups with added wild bitter gourd than those of the remaining groups. Blood lipids (TG, cholesterol, and NEFA) were also lower in comparison to the sepsis group, and blood glucose concentrations recovered and approached normal levels. Blood biochemistry values related to inflammation reactions indicated GOT, GPT, C-RP, and NOconcentrations of groups with added wild bitter gourd were all lower than those of the sepsis group. Secretion levels of the spleen pro-inflammatory cytokines IL-1, IL-6, and TNF-α tallied significantly lower in comparison to the sepsis group, whereas secretion levels of IL-10 anti-inflammatory cytokine increased. Expression level of proteins NF-κB, iNOS, and COX-2 were significantly inhibited. Results indicate wild bitter gourd in diets promoted lipid metabolism, reducing fat accumulation, and improving low blood glucose in sepsis. Addition of wild bitter gourd can reduce inflammation biochemical markers or indicators and pro-inflammatory cytokines in the body, hence improving the inflammation responses in mice with sepsis.

## 1. Introduction

Wild bitter gourd (*Momordica charantia* L. var. *abbreviate* Seringe), a *Momordica charantia* variety, is consumed as both as a vegetable and folk medicine in Taiwan [1,2]. The most noticeable pharmacological property of wild bitter gourd (WBG) is its hypoglycemic activity, not only used widely in herbal medicine but also demonstrated in rodent models [3] and Type 2 diabetic patients [4]. WBG is known to contain numerous components: e.g., alkaloids, steroidal glucosides, phenolics [5], lysophosphatidylcholines [6], conjugated linolenic acid isomers [7], and cucurbitane-type triterpenoids [8,9]. WBG is considered more potent in disease prevention than bitter gourd, yet little is known about WBG’s biological and physiological characteristics. Its anti-inflammation property is thus linked to categorization as a “cooling food” in traditional/folk medicine [10]. Precise mechanisms and well-controlled clinical trials of its anti-inflammation effects have not been completely delineated [11].

Inflammation reactions are closely related to progression of and tissue damage caused by numerous diseases, making research on anti-inflammatory effects crucial. Sepsis pathogens notably relate to lipopolysaccharides (LPS) (a.k.a. endotoxins) released by Gram-negative bacteria cell walls. Body cells are stimulated by microbial components. Through production of cytokines and tumor necrosis factor-α (TNF-α), a series of complements, white blood cells, and vascular endothelial cells are activated. Numerous microorganisms produce hypervirulent exotoxins that directly damage human tissue [12]. Inflammation process involves activation of macrophages, inciting expression of cyclooxygenase-2 (COX-2), and leading synthesis of the inflammatory mediator prostaglandin E_2_ (PGE_2_). Prostaglandins produced primarily by macrophages are critical pro-inflammatory mediators [13]. If the formation of inflammatory mediators is reduced, discomfort caused by inflammation is alleviated [14]; this is the purpose of anti-inflammatory drugs.

Peroxisome proliferator-activated receptors (PPARs) are regarded as therapeutic targets for cardiovascular disease: activation of these not only directly regulates genes of vascular and inflammatory cells involved in atherosclerosis, but also indirectly promotes glucose utilization and serum lipid profiles [15,16]. Prior research in our laboratory pointed to bitter gourd components that activate PPARα and PPARγ [1]. Various bitter gourds (green, white, pearl, wild) on the market in Taiwan were compared by *in vitro* tests to find the wild variety with greatest anti-inflammatory capacity. In addition, anti-inflammatory capacities of different sections of this gourd (fruit, seeds, leaves, stems, flowers) were contrasted. The fruit section most effectively inhibited inflammatory mediators PGE_2_ and nitric oxide (NO) [9,10,11]; we probed anti-inflammatory effects of wild bitter gourd fruit in *in vivo* animal inflammation models (sepsis). We noted influence of bitter melon on animal models with acute septicemia while examining possible mechanisms of action and effect.

## 2. Results and Discussion

Our previous study had already assessed and compared the survival rate in sepsis (i.p. LPS 15 mg/kg) and treatment groups (L, M, H), every 12 h for 120 h. By COX’s proportion hazards regression test, the treatment groups had significantly lower hazard ratios compared with the sepsis group (*p* = 0.037). The survival rate of treatment groups (L, M, H) were significantly greater than that of sepsis group (S) (*p* < 0.05). Most studies use low dose LPS (5 mg/kg) for cytokine profile and high doses (35–44 mg/kg) for survival studies. The current study utilized a sub-lethal dose of 15 mg/kg according to a dose titration study showing mice with 100% survival at 14 h and 50% survival at 24 h after LPS challenge, which allows for examining the profile of cytokines not only at the early, but also at the late stage of LPS-induced acute inflammation.

Table 1 shows changes in body weight and intake level for each group: body weights of Groups L, M, and H significantly lower than those of Groups N, S, and P. Group H had most significant weight-loss effect. Organ lesions caused by sepsis spawn metabolic abnormalities in blood lipids (TG, NEFA, and cholesterol) within the body. This is significantly reduced after adding wild bitter gourd, trending toward a dose response. Table 2 shows that the blood glucose abnormalities of Group S followed low blood glucose situations. This may be caused by multiple organ failure (MOF) following sepsis and is considered a complication thereof [17,18]. Wild bitter gourd is known to activate PPARα, thus moderating lipid metabolism [14,19]; its active components, such as conjugated linoleic acid (CLA), are speculated to activate PPARα, thereby facilitating β-oxidation for fatty acids and maintaining constancy of lipid metabolism within cells [20,21]. PPARα also regulates ketogenesis within the body, thereby moderating metabolism and balance of lipids [21,22].

**Table 1:** Initial body weight, final body weight, and food intake for mice.

Group	n	Body Weight		Food Intake
		Initial BW (g)	Final BW (g)	(g/day)
**N**	12	22.6 ± 2.0 ^a^	25.8 ± 1.7 ^b^	3.8 ± 0.2 ^a^
**S**	12	23.0 ± 1.5 ^a^	27.0 ± 1.3 ^a^	3.8 ± 0.3 ^a^
**L**	12	23.3 ± 1.0 ^a^	23.5 ± 0.9 ^c^	3.9 ± 0.3 ^a^
**M**	12	22.8 ± 1.3 ^a^	22.8 ± 0.5 ^c^	3.9 ± 0.3 ^a^
**H**	12	23.6 ± 1.3 ^a^	19.8 ± 1.5 ^d^	3.9 ± 0.6 ^a^
**P**	12	23.0 ± 0.7 ^a^	25.2 ± 0.5 ^a^	3.8 ± 0.2 ^a^

Values not sharing letters in the same vertical column differ significantly from one another by one-way ANOVA and Duncan^’^s Multiple Range Test (*p* < 0.05) among six groups. N: Normal group, S: Sepsis group (i.p. LPS), L: Sepsis group with low-dose WBG, M: Sepsis group with moderate-dose WBG, H: Sepsis group with high-dose WBG, P: Positive control group (i.p. PDTC).

**Table 2:** Serum triglyceride, cholesterol, NEFA, and glucose concentrations of LPS-induced sepsis mice.

Group	n	Triglyceride (mg/dL)	Cholesterol (mg/dL)	NEFA (mmol/L)	Glucose (mg/dL)
**N**	12	186.8 ± 11.7 ^a^	194.7 ± 20.3 ^c^	0.64 ± 0.03 ^d^	102.6 ± 4.8 ^a^
**S**	12	224.2 ± 10.9 ^bc^	227.1 ± 36.0 ^a^	0.95 ± 0.05 ^a^	50.1 ± 5.2 ^c^
**L**	12	196.0 ± 6.6 ^c^	222.9 ± 15.5 ^b^	0.92 ± 0.01 ^a^	50.6 ± 4.9 ^c^
**M**	12	197.3 ± 23.9 ^c^	222.1 ± 24.2 ^a^	0.85 ± 0.01 ^a^	72.7 ± 16.4 ^b^
**H**	12	188.7 ± 21.4 ^d^	207.1 ± 13.8 ^c^	0.83 ± 0.01 ^b^	64.5 ± 2.1 ^b^
**P**	12	186.4 ± 7.6 ^a^	202.9 ± 22.2 ^b^	0.79 ± 0.01 ^c^	101.4 ± 9.4 ^a^

Values not sharing letters in the same vertical column differ significantly from one another by one-way ANOVA and Duncan^’^s Multiple Range Test (*p* < 0.05) among six groups. N: Normal group, S: Sepsis group (i.p. LPS), L: Sepsis group with low-dose WBG, M: Sepsis group with moderate-dose WBG, H: Sepsis group with high-dose WBG, P: Positive control group (i.p. PDTC).

Earlier studies confirm wild bitter gourd as containing PPARα and PPARγ activators [1] that facilitate lipid metabolism, reduce blood lipids, and cause anti-inflammatory activity [2]. This subsequently reduces fat accumulation, resulting in weight-loss effect. Besides reducing blood glucose concentrations, PPARγ activation reduces inflammation reactions. Molecular mechanisms may entail inhibition of cytokines secreted by monocytes following PPARγ activation [14]. This interferes with transmission of messages for NF-κB, signal transducers and activators of transcription (STAT), activator protein-1 (AP-1), *etc.*, inhibiting expression of inflammatory genes like IL-1, IL-2, IL-6, IL-8, TNF-α, and metalloproteinase (MMPs) [23]. PPAR agonists currently treat atherosclerosis; they activate both receptors simultaneously and are regarded as having greatest potential. Whether significant reduction in body weight of mice fed 10% wild bitter gourd wreaks negative physiological effects merits further research.

**Figure 1:**
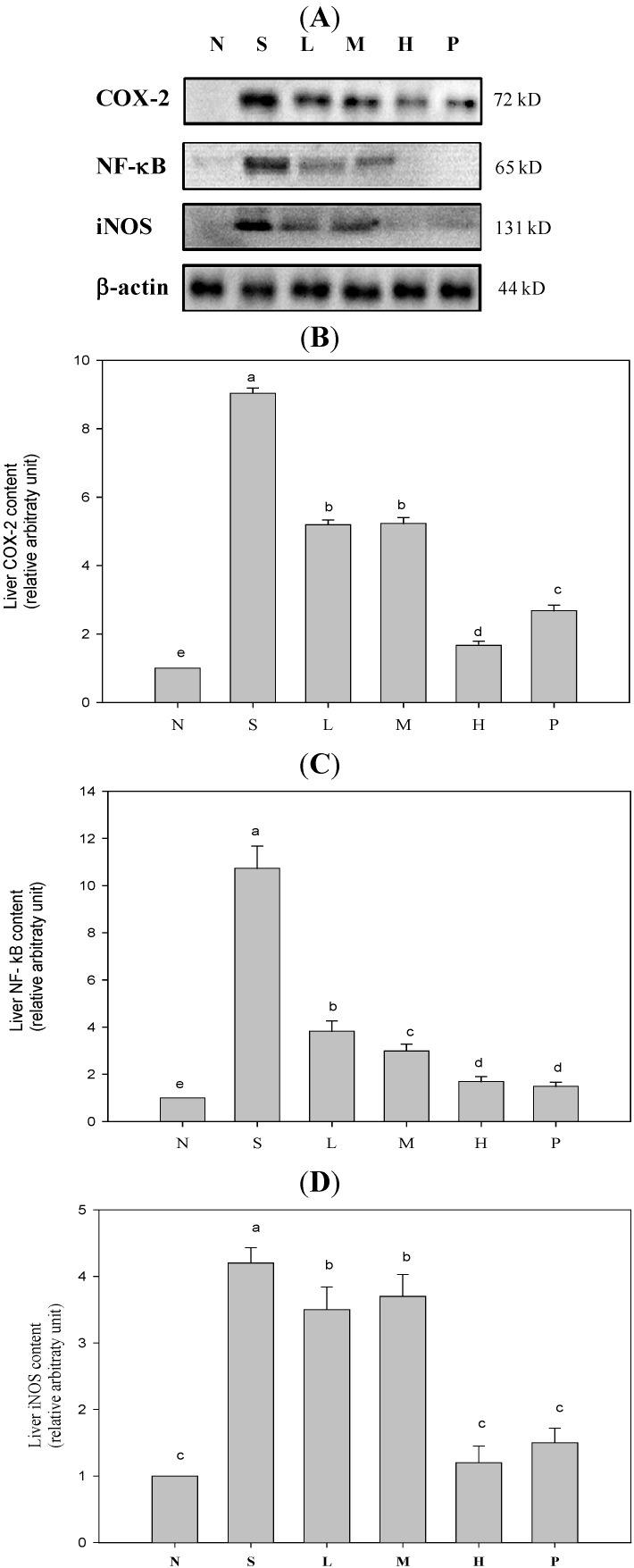
COX-2, NF-κB, and iNOS inflammatory protein expression of LPS-induced sepsis mice. (**A**) The western blotting results of COX-2, NF-κB, and iNOS. (**B**) The immunoblot quantitative result of COX-2 protein. (**C**) The immunoblot quantitative result of NF-κB protein. (**D**) The immunoblot quantitative result of iNOS protein. Values are the mean ± SD of each group. Values not sharing a superscript (a–e) differ significantly by one-way ANOVA and Duncan’s Multiple Range Test (*p* < 0.05) among groups. One representative immunoblot from three independent experiments is shown. N: Normal group, S: Sepsis group (i.p. LPS), L: Sepsis group with low-dose WBG, M: Sepsis group with moderate-dose WBG, H: Sepsis group with high-dose WBG, P: Positive control group (i.p. PDTC).

Figure 1 shows expression levels of proteins COX-2, iNOS, and NF-κB (phosphorylated form) increasing significantly within the body during the inflammation reaction process, thus elevating concentrations of downstream inflammatory mediators like PGE_2_ and NO (data not shown). Table 3 plots pro-inflammatory cytokine (TNF-α, IL-1β, and IL-6) concentrations in spleens of mice with sepsis as definitely higher than those in Group N. These ratios dropped sharply after wild bitter gourd was added to diets. Secretions of anti-inflammatory cytokine IL-10 increased, presenting dose response trends. Serum inflammatory mediators like NO, AST, ALT, and C-RP, all fell significantly in mice after four weeks of test feeds containing various doses of wild bitter gourd, consequently reducing damage to organs and tissue. Group H had most significant results, falling to values equal to those of Group N (Table 4). When organs are damaged, AST (GOT) and ALT (GPT) within cells are released into serum. Extant literature indicates that after sepsis occurs, cell necrosis increases during the septic process, releasing more liver cell enzymes. AST and ALT concentrations in blood rise [24], causing inflammation response accompanied by severe organ damage [25].

**Table 3:** Spleen level of inflammatory cytokines concentrations of LPS-induced sepsis mice.

Group	n	TNF-α (µg/mL)	IL-1β (µg/mL)	IL-6 (µg/mL)	IL-10 (µg/mL)
**N**	12	16.68 ± 0.20 ^bc^	18.70 ± 0.77 ^c^	17.64 ± 0.33 ^d^	20.40 ± 0.33 ^d^
**S**	12	20.65 ± 0.43 ^a^	20.78 ± 0.78 ^b^	23.68 ± 1.26 ^a^	22.38 ± 0.48 ^c^
**L**	12	15.72 ± 1.25 ^d^	18.55 ± 0.78 ^c^	21.20 ± 0.91 ^c^	22.48 ± 0.18 ^c^
**M**	12	17.53 ± 0.36 ^b^	21.46 ± 0.34 ^ab^	22.71 ± 1.80 ^ab^	23.54 ± 0.25 ^a^
**H**	12	14.37 ± 0.93 ^e^	16.38 ± 1.76 ^d^	21.93 ± 0.37 ^bc^	23.42 ± 0.32 ^ab^
**P**	12	16.57 ± 0.45 ^cd^	22.09 ± 0.13 ^a^	18.71 ± 0.56 ^d^	23.05 ± 0.31 ^b^

Values not sharing letters in the same vertical column differ significantly from one another by one-way ANOVA and Duncan’s Multiple Range Test (*p* < 0.05) among six groups. N: Normal group, S: Sepsis group (i.p. LPS), L: Sepsis group with low-dose WBG, M: Sepsis group with moderate-dose WBG, H: Sepsis group with high-dose WBG, P: Positive control group (i.p. PDTC).

**Table 4:** Serum inflammatory mediator concentrations of LPS-induced sepsis mice.

Group	n	NO (µM)	AST(GOT) (unit/L)	ALT(GPT) (unit/L)	C-RP (ng/mL)
**N**	12	23.8 ± 1.6 ^c^	27.1 ± 2.4 ^c^	31.3 ± 2.1 ^a^	167.0 ± 18.1 ^b^
**S**	12	39.3 ± 5.1 ^a^	48.9 ± 3.8 ^a^	46.6 ± 2.5 ^b^	227.0 ± 20.0 ^a^
**L**	12	33.5 ± 2.7 ^b^	43.1 ± 2.3 ^b^	40.2 ± 3.0 ^c^	211.5 ± 10.4 ^a^
**M**	12	25.7 ± 1.6 ^b^	40.5 ± 2.6 ^b^	36.3 ± 2.1 ^d^	220.9 ± 19.9 ^a^
**H**	12	24.7 ± 1.5 ^c^	25.0 ± 2.3 ^c^	27.4 ± 2.3 ^a^	181.4 ± 16.7 ^b^
**P**	12	25.0 ± 3.5 ^c^	16.6 ± 3.1 ^d^	19.6 ± 2.4 ^e^	188.5 ± 17.9 ^b^

Values not sharing letters in the same vertical column differ significantly from one another by one-way ANOVA and Duncan’s Multiple Range Test (*p* < 0.05) among six groups. N: Normal group, S: Sepsis group (i.p. LPS), L: Sepsis group with low-dose WBG, M: Sepsis group with moderate-dose WBG, H: Sepsis group with high-dose WBG, P: Positive control group (i.p. PDTC).

LPS is currently known to increase transcriptional effects of iNOS mRNA by activating NF-κB [26]. Two hours following acute attacks of sepsis, NO is produced excessively within kidneys, resulting in low blood pressure and tachycardia [27]. Extant literature indicates that activation of PPARγ can inhibit the formation of inflammatory mediators like TNF-α, IL-1β, and IL-6 [28]. PPARγ also reduces iNOS activity by inhibiting transcription factor NF-κB, thence alleviating inflammation [29]. This study indicates 10% wild bitter gourd diet significantly inhibiting expression of iNOS protein, reducing formation of inflammatory mediator NO. Likewise, adding wild bitter gourd to diets can reduce formation of inflammatory mediator PGE_2_ by reducing expression of COX-2 protein. This reduces production of pro-inflammatory cytokines and other substances. Anti-inflammatory cytokine IL-10 also rises significantly, achieving anti-inflammatory effects. Extant literature indicates butanol-soluble fraction of its placenta extract strongly suppressing LPS-induced TNF-α yield in RAW264.7 cells. Anti-inflammatory components were identified as 1-α-linolenoyl-lysophosphatidylcholine (LPC), 2-α-linolenoyl-LPC, 1-lynoleoyl-LPC, and 2-linoleoyl-LPC [6].

In Groups L, M, and H, since wild bitter gourd contains substances capable of activating PPARγ, severity of septic inflammation reaction was retarded, thus reducing organ damage. Extant literature indicates liver vascular sinus accumulating formation of substantial amounts of platelets, red blood cells, white blood cells, and microthrombosis during endotoxic shock. This causes expansion and obstruction to the vascular sinus, thereby damaging liver function [30]. Acute septicemia causes substantial increases in vascular permeability and rapid heart rate. The ensuing insufficient return of blood to organs affects heart weight and blood volume. This study also indicates that, regarding weights of organs (liver, kidneys, and spleen), Group H proves able to reduce organomegaly (data not shown) compared with Group S. These will help design human studies necessary to prove efficacy of bitter gourd against clinical sepsis.

## 3. Experimental Section

### 3.1. Preparation of Test Samples

We homogenized fresh wild bitter gourd (whole fruits including seeds) into a pulp. After using gauze for filtering, we freeze-dried the residue and ground it into powder. For the basic components of the feed, we referenced the AIN-76 formula and made appropriate adjustments, adding various ratios of wild bitter gourd lyophilized powder and dietary fiber. We adjusted dietary fiber content for groups to identical ratios. We added sterile water to shape prepared powder feed into pellets (4 g for each pellet), refrigerating these at −20 °C.

### 3.2. Animal Treatment and Grouping

Our 72 six-week-old male BALB/c mice (body weight approximately 16–19 g) were purchased from the National Laboratory Animal Center. They were first fed a rodent chow diet for one week of adaptation. When their body weights averaged approximately 22 g, we randomly divided them based on their body weights. Mice were fed for a total of four weeks during the experiment. Feed intake levels during experimentation period were recorded daily, body weight recorded weekly. Each mouse was fed 4 g/day test feed. Excess supply of drinking water was used to allow free intake.

This study used the endotoxin model to induce acute septicemia reactions. Before mice sacrifice, other than the normal (N) group, each group received intraperitoneal injections of LPS. The LPS-induced sepsis mice was made by injecting 15 mg/kg BW of LPS (*E. coli* O127: B8; Sigma, St. Louis, MO, USA) in 0.4 mL normal saline intraperitoneally (i.p.). The positive control (P) group received intravenous injections of anti-inflammatory drug pyrrolidinedithiocarbamic acid ammonium salt (PDTC) 1 h before the LPS injection. The normal control group was injected with normal saline. Mice were fed for different treatment diet for 4 weeks, and then each group received intraperitoneal injections of LPS (15 mg/kg), except the normal (N) group. Mice in the PDTC group were injected i.p. with PDTC (50 mg/kg), a dose with anti-inflammatory effects as reported, 1 h before LPS challenge. After 4 weeks of treatment, mice were sacrificed by decapitation and the blood was removed.After packaging blood and organs obtained, these were cryopreserved in refrigerators at −80 °C for later analysis. Research accorded with internationally accepted principles for laboratory animal use and care, as reviewed and approved by the Institutional Animal Care and Use Committee Guidelines of China Medical University (IACUC, protocol No: 97-26-N).

Six experimental groups were used, each identified by the names and symbols indicated below:

(1) N: Normal group (i.p. normal saline), fed the chow diet.
(2) S: Sepsis group (i.p. LPS, 15 mg/kg BW), fed the chow diet.
(3) L: Sepsis group with low-dose (1%) wild bitter gourd lyophilized powder added, fed feed including 1% wild bitter gourd lyophilized powder.
(4) M: Sepsis group with moderate-dose (2%) wild bitter gourd lyophilized powder added, fed feed including 2% wild bitter gourd lyophilized powder.
(5) H: Sepsis group with high-dose (10%) wild bitter gourd lyophilized powder added, fed feed including 10% wild bitter gourd lyophilized powder.
(6) P: Positive control group (i.p. PDTC, 50 mg/kg BW), fed the chow diet.

### 3.3. Blood Preparation and Biochemical Assay

After 4 weeks’ treatment, mice were fasted overnight (12 h) and decapitated. Blood was centrifuged at 1700× *g* at 4 °C for 30 min to separate serum gauged by enzymatic methods, using commercial kits (Randox Lab, Crumlin, Northland, UK) for TG, cholesterol, glucose and non-esterified fatty acid (NEFA), these biochemical analysis as previously described [1]. Serum inflammatory mediator included NO, glutamate oxaloacetate transaminase (GOT), glutamate pyruvate transaminase (GPT) and C-reactive protein (C-RP) concentration were analyzed by clinical test kits (Roche Cobas Mira plus, Berlin, Germany).

### 3.4. Measurement of Splenocyte Cytokine Levels by ELISA

Spleens were isolated from sacrificed mice, tissues were excised and weighed. A portion of fresh spleens were homogenized in 50 mmol/L Tris-HCl buffer (pH 8.3) and centrifuged at 600× *g* for 6 min. The splenocyte suspension (4 × 10^6^ cells/well) cells were stimulated with ConA (5 µg/mL) performed in 48-well round-bottom plates. The production of cytokines TNF-α, IL-1β, IL-6, and IL-10 in the cell supernatants were collected for cytokine assay after 48-h incubation, assayed by commercial cytokine ELISA kit. The data were calculated according to the cytokine standard curve. Splenocyte cytokine levels were determined by commercially available enzyme-linked immunosorbent assay (ELISA) kit (Bio-source International Inc., Camarillo, CA, USA) according to the manufacturer’s instructions. Cytokines TNF-α, IL-1β, IL-6 and IL-10 were determined by a standard curve, concentrations were expressed as µg/mL.

### 3.5. Western Blot Analysis

Liver tissues were homogenized in lysis buffer (0.6% NP-40, 150 mM NaCl, 10 mM HEPES (pH 7.9), 1 mM EDTA, and 0.5 mM PMSF) at 4 °C. Fifty microgrammes of protein subjected to 10% SDS-PAGE was transferred to polyvinylidene fluoride (PVDF) membrane (NEN Life Science, Boston, MA, USA), blot immunodetected with enhanced chemiluminescence (ECL) Western blot kit (Amersham International, Amersham, UK) in which goat anti-mouse COX-2, nuclear factor-kappaB (NF-κB), inducible nitric oxide synthase (iNOS) and β-actin antiserum served as primary antibody (Millipore, Billerica, MA, USA) and goat anti-rabbit IgG-biotinylated species-specific whole antibody (Amersham Biosciences, Piscataway, NJ, USA) was used as secondary antibody. Blots were quantified by relative intensity compared to control with Kodak Molecular Imaging Software (Version 4.0.5, Eastman Kodak Company, Rochester, NY, USA), represented in relative intensities.

### 3.6. Statistical Analysis

Our experimental results indicate mean ± SD. After confirming normal distribution, we employed one-way ANOVA and Duncan’s multiple range test to identify differences between independent sample groups, using SPSS 13.0 software (SPSS Inc., Chicago, IL, USA) for statistical analysis.

## 4. Conclusions

Wild bitter gourd in diets facilitates lipid metabolism, reducing blood lipid concentration and body weight. Group H shows most significant results. Adding wild bitter gourd to diets of sepsis-induced mice reduced expression of proteins COX-2, iNOS, and NF-κB, all associated with inflammation. It reduced secretions of pro-inflammatory cytokines and other substances, hence improved the inflammation responses in sepsis mice. Its numerous health benefits include reducing blood lipids, improving blood glucose, and combating inflammation. In the future, with active ingredients purified, isolated, and identified, this common crop can be used clinically as nutritional supplement to improve acute endotoxin-induced septicemia.

## Figures and Tables

**Figure A1:**
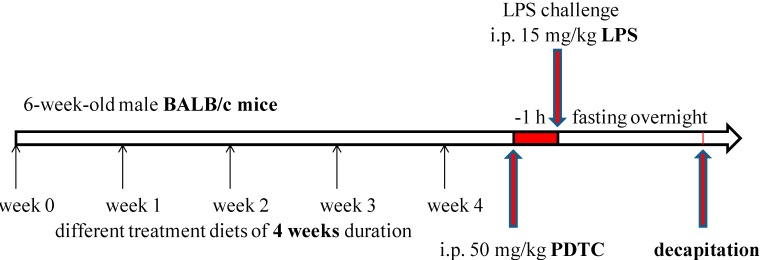
Experiment Process of Time Line.

## Abbreviation

WBG: Wild bitter gourd
LPS: Lipopolysaccharides
COX-2: Cyclooxygenase-2
TNF-α: Tumor necrosis factor-α
PGE_2_: Prostaglandin E_2_
PPARs: Peroxisome proliferator-activated receptors
NO: Nitric oxide
PDTC: Pyrrolidinedithiocarbamic acid ammonium salt
NEFA: Non-esterified fatty acid
GOT: Glutamate oxaloacetate transaminase
C-RP: C-reactive protein
GPT: Glutamate pyruvate transaminase
PVDF: Polyvinylidene fluoride
ECL: Enhanced chemiluminescence
NF-κB: Nuclear factor-kappaB
iNOS: Inducible nitric oxide synthase
MOF: Multiple organ failure
CLA: Conjugated linoleic acid
STAT: Signal transducers and activators of transcription
LPC: Lysophosphatidylcholine
AP-1: Activator protein-1
MMPs: Metalloproteinase

## Appendix

This figure (time line) was supplement of *3.2. Animal Treatment and Grouping* to explain the animal experiment process.

## References

[1] Chao C.Y., Yin M.C., Huang C.J. (2011). Wild bitter gourd extract up-regulates mRNA expression of PPARα, PPARγ and their target genes in C57BL/6J mice. J. Ethnopharmacol..

[2] Lii C.K., Chen H.W., Yun W.T., Liu K.L. (2009). Suppressive effects of wild bitter gourd (Momordica charantia Linn. var. abbreviate ser.) fruit extracts on inflammatory responses in RAW264.7 macrophages. J. Ethnopharmacol..

[3] Uebanso T., Arai H., Taketani Y., Fukaya M., Yamamoto H., Mizuno A., Uryu K., Hada T., Takeda E. (2007). Extracts of *Momordica charantia* suppress postprandial hyperglycemia in rats. J. Nutr. Sci. Vitaminol..

[4] Welihinda J., Karunanayake E.H., Sheriff M.H. (1986). Effect of *Momordica charantia* on the glucose tolerance in maturity onset diabetes. J.Ethnopharmacol..

[5] Krawinkel M.B., Keding G.B. (2006). Bitter gourd (*Momordica charantia*): A dietary approach to hyperglycemia. Nutr. Rev..

[6] Kobori M., Nakayama H., Fukushima K., Ohnishi-Kameyama M., Ono H., Fukushima T., Akimoto Y., Masumoto S., Yukizaki C., Hoshi Y. (2008). Bitter gourd suppresses lipopolysaccharide-induced inflammatory responses.. J. Agric. Food Chem..

[7] Chuang C.Y., Hsu C., Chao C.Y., Wein Y.S., Kuo Y.H., Huang C.J. (2006). Fractionation and identification of 9c, 11t, 13t-conjugated linolenic acid as an activator of PPARalpha in bitter gourd (Momordica charantia L.). J. Biomed. Sci..

[8] Chang C.I., Tseng H.I., Liao Y.W., Yen C.H., Chen T.M., Lin C.C., Cheng H.L. (2011). *In vivo* and in vitro studies to identify the hypoglycaemic constituents of Momordica charantia wild variant WB24. Food Chem..

[9] Hsu C., Hsieh C.L., Kuo Y.H., Huang C.J. (2011). Isolation and identification of cucurbitane-type triterpenoids with partial agonist/antagonist potential for estrogen receptors from *Momordica charantia*. J. Agric. Food Chem..

[10] Hsu C., Tsai T.H., Li Y.Y., Wu W.H., Huang C.J., Tsai P.J. (2012). Wild bitter melon (*Momordica charantia* Linn. var. *abbreviata* Ser.) extract and its bioactive components suppress *Propionibacterium acnes*-induced inflammation. Food Chem..

[11] Leung L., Birtwhistle R., Kotecha J., Hannah S., Cuthbertson S. (2009). Anti-diabetic and hypoglycaemic effects of *Momordica charantia* (bitter melon): A mini review. Brit. J. Nutr..

[12] Weinberg J.B. (2000). Nitric oxide synthase and cyclooxygenase 2 interactions in inflammation. Immunol. Res..

[13] Nathan C.F. (1987). Secretory products of macrophages. J. Clin. Investig..

[14] Chao C.Y., Huang C.J. (2003). Bitter gourd (*momordica charantia*) extract activates peroxisome proliferator-activated receptors and upregulates the expression of the acyl CoA oxidase gene in H4IIEC3 hepatoma cells. J. Biomed. Sci..

[15] Chinetti G., Fruchart J.C., Staels B. (2000). Peroxisome proliferator-activated receptors (PPARs): nuclear receptors at the crossroads between lipid metabolism and inflammation. Inflamm. Res..

[16] Chung J.H., Seo A.Y., Chung S.W., Kim M.K., Leeuwenburgh C., Yu B.P., Chung H.Y. (2008). Molecular mechanism of PPAR in the regulation of age-related inflammation. Ageing Res. Rev..

[17] Reddy R.C. (2008). Immunomodulatory role of PPAR-gamma in alveolar macrophages. J. Investig. Med..

[18] Maitra S.R., Wojnar M.M., Lang C.H. (2000). Alterations in tissue glucose uptake during the hyperglycemic and hypoglycemic phases of sepsis. Shock.

[19] Huang C.J., Wu M.C. (2002). Differential effects of foods traditionally regarded as ‘heating’ and ‘cooling’ on prostaglandin E2 production by a macrophage cell line. J. Biomed. Sci..

[20] Lee S.S., Pineau T., Drago J., Lee E.J., Owens J.W., Kroetz D.L., Fernandez-Salguero P.M., Westphal H., Gonzalez F.J. (1995). Targeted disruption of the alpha isoform of the peroxisome proliferator-activated receptor gene in mice results in abolishment of the pleiotropic effects of peroxisome proliferators. Mol. Cell. Biol..

[21] Yumiko Y., Masashi H., Takehiko S., Rikako S., Satoru O., Hiroyuki K., Takuji T., Kazuo M. (2005). Bitter gourd seed fatty acid rich in 9*c*,11*t*,13*t*-conjugated linolenic acid induces apoptosis and up-regulates the GADD45, p53 and PPARγ in human colon cancer Caco-2 cells. Prostaglandins Leukot. Essent. Fatty Acids.

[22] Rodriguez J.C., Gil-Gomez G., Hegardt F.G., Haro D. (1994). Peroxisome proliferator-activated receptor mediates induction of the mitochondrial 3-hydroxy-3-methylglutaryl-CoA synthase gene by fatty acids. J. Biol. Chem..

[23] Mukherjee R., Jow L., Noonan D., McDonnell D.P. (1994). Human and rat peroxisome proliferator activated receptors (PPARs) demonstrate similar tissue distribution but different responsiveness to PPAR activators. J. Steroid Biochem..

[24] Yukihiro S. (2008). Liver in systemic disease. World J. Gastroenterol..

[25] Surh Y.J., Chun K.S., Cha H.H., Han S.S., Keum Y.S., Park K.K., Lee S.S. (2001). Molecular mechanisms underlying chemopreventive activities of anti-inflammatory phytochemicals: Down-regulation of COX-2 and iNOS through suppression of NF-kappa B activation. Mutat. Res..

[26] Bogdan C. (2001). Nitric oxide and the immune response. Nat. Immunol..

[27] Gardiner S.M., Kemp P.A., March J.E., Bennett T. (1995). Cardiac and regional haemodynamics, inducible nitric oxide synthase (iNOS) activity, and the effects of NOS inhibitors in conscious, endotoxaemic rats. Brit. J. Pharmacol..

[28] Hanada T., Yoshimura A. (2002). Regulation of cytokine signaling and inflammation. Cytokine Growth Factor Rev..

[29] Ricote M., Li A.C., Willson T.M., Kelly C.J., Glass C.K. (1998). Peroxisome proliferator-activated receptor-gamma is a negative regulator of macrophage activation. Nature.

[30] Iwasashi H., Suzuki M., Unno M., Utiyama T., Oikawa M., Kondo N., Matsuno S. (2003). Inhibition of heme oxygenase ameliorates sepsis-induced liver dysfunction in rats. Surg. Today.

